# Risk Management of Clinical Reference Dosimetry of a Large Hospital Network Using Statistical Process Control

**DOI:** 10.4236/ijmpcero.2021.103011

**Published:** 2021-07-30

**Authors:** Seng-Boh Lim, Thomas LoSasso, Maria Chan, Laura Cervino, Dale Michael Lovelock

**Affiliations:** Department of Medical Physics, Memorial Sloan Kettering Cancer Center, New York, USA

**Keywords:** TG-51, Dosimetry, Process Control, Risk Management, Large Hospital Network

## Abstract

Managing TG-51 reference dosimetry in a large hospital network can be a challenging task. The objectives of this study are to investigate the effectiveness of using Statistical Process Control (SPC) to manage TG-51 workflow in such a network. All the sites in the network performed the annual reference dosimetry in water according to TG-51. These data were used to cross-calibrate the same ion chambers in plastic phantoms for monthly QA output measurements. An energy-specific dimensionless beam quality cross-calibration factor, $k_{qn}^{SW}$, was derived to monitor the process across multiple sites. The SPC analysis was then performed to obtain the mean, $\langle k_{qn}^{SW}\rangle$, standard deviation, *σ*_*k*_, the Upper Control Limit (UCL) and Lower Control Limit (LCL) in each beam. This process was first applied to 15 years of historical data at the main campus to assess the effectiveness of the process. A two-year prospective study including all 30 linear accelerators spread over the main campus and seven satellites in the network followed. The ranges of the control limits (±3*σ*) were found to be in the range of 1.7% – 2.6% and 3.3% – 4.2% for the main campus and the satellite sites respectively. The wider range in the satellite sites was attributed to variations in the workflow. Standardization of workflow was also found to be effective in narrowing the control limits. The SPC is effective in identifying variations in the workflow and was shown to be an effective tool in managing large network reference dosimetry.

## 1. Introduction

The healthcare network has been seen significant expansion in recent years. Managing and monitoring the quality of the radiation delivery systems in a large expanding network can be an expensive and challenging task; new staff and equipment might be acquired through mergers or acquisitions, for example, to meet the targeted capacity within a defined time frame.

AAPM has published various Task Group (TG) reports, such as TG-142 [1], TG-51 [2], and MPPG#8 [3], that provide Quality Assurance (QA) recommendations of treatment machines to clinical physicists. Even with the process simplification in TG-51 from the earlier TG-21 [4], clinical reference dosimetry arguably still involves many steps that can potentially lead to systematic errors [5]. In hospitals with similar treatment machines, a standard set of energy-specific beam models, such as Varian’s Golden Beam Data [6] for Clinac’s or Representative Beam Data [7] for TrueBeam’s (Varian, Palo Alto, CA, USA), is often used for multiple machines in the treatment planning system in order to mitigate the effort and risk of maintaining multiple beam models. Adequate resources, however, are still required to maintain tight monitoring, and known tolerances as individual machines may deviate from the standard model in varying degrees. The Imaging and Radiation Oncology Core (IROC) Remote Dosimetry Program, utilizes mailed Optical Stimulated Luminescence Detectors (OSLD) which have a standard deviation calibration uncertainty [8] of 1.6%, to provides a valuable independent dose verification, and is considered the standard for monitoring of the reference dosimetry accuracy within and among institutions. A parallel internal quality control mechanism to monitor the variability of the reference dosimetry would improve the quality and safety of the process within a large hospital network [9].

Statistical Process Control (SPC) [10] [11] is the application of statistical tools to control, monitor, and improve a process. One of the important aspects of SPC is to characterize the random variation of a process in order to establish the action-level thresholds. Through this exercise, the occurrence and magnitude of systematic errors can be identified and minimized [10] [11] [12], reducing the variability of the underlying component processes. Pawlicki [13] reported on applying SPC to the performance of daily linear accelerator (Linac) QA output, flatness and symmetry measurements for a photon beam. Subsequently, SPC was applied in the longitudinal monitoring of Patient-Specific QA (PSQA) in various modalities [14] [15]. The quality control method was recently expanded to real-time transit dosimetry [16] where a large amount of real-time EPID images were used for PSQA. Recently, SPC was also investigated in the monitoring of the longitudinal performance of a treatment machine [17]. However, the cross-sectional QA monitoring of multiple machines using SPC has not been the focus to improve the quality of clinical reference dosimetry within a large hospital network. The objectives of this present work are to utilize SPC to: 1) identify efficient metrics that correlate with variability in the TG-51 process; 2) specify the control limits of the variable; 3) demonstrate a reduction of the variability in the TG-51 process.

In this paper, we report a risk management method on clinical reference dosimetry using SPC for a large hospital network. Following the introduction, an overview of reference dosimetry, retrospective and prospective analysis of the control process will be presented.

## 2. Method

There are currently eight campuses with 30 Linacs in the network, 11 at the main campus and 19 at the satellites. All the Linacs are currently Varian (Varian Medical Systems, Palo Alto, CA) including 22 TrueBeam, 6 Clinac, and 2 6EX type Linacs. A summary of the machine type distribution at the main and the satellite sites is listed in Table 1.

The main site has a less homogeneous machine type with about 64% being TrueBeam type while the satellite sites have about 79% being TrueBeam.

The reference dosimetry at all sites is performed annually in water in accordance with AAPM TG-51 [2] and TG-51 addendum [5]. All reference doses, *D*_*ref*_, were measured with Accredited Dosimetry Calibration Laboratory (ADCL) calibrated A12 (Standard Imaging, WI) Farmer-type chambers and ADCL calibrated Max 4000 (Standard Imaging, WI) electrometers. All beam outputs are measured with a field size of 10 × 10 cm^2^, at source to surface distance (SSD) of 100 cm and depth of 10 cm in water. *D*_*ref*_, is given by:

(1) $$D_{ref}(10)=k_{q}N_{w}^{c}M_{w}$$

where *k*_*q*_, $N_{w}^{c}$, and *M*_*w*_ are the beam quality factor, ADCL calibration factor, and corrected measurements in water [2]. The dosimetry is repeated in Solid Water (Sun Nuclear Corp, Melbourne, FL) on the same day with the same A12 chamber and Max 4000 electrometer used for TG51, as well as a second A12 and Max 4000 combination to be used for monthly QA, at the depth of $d_{cal}^{SW}$ in Solid Water^®^ (Sun Nuclear Corp, Melbourne, FL) as shown in Table 2.

These depths were chosen to minimize setup variations between energies while maintaining less than 0.5% deviation with measured dose at the true depth of maximum dose, dmax; they are based on averages for the 10 Varian Linacs at the main campus. Photon and electron doses in the solid phantom were measured with the field size of 10 × 10 cm^2^, source to axis distance (SAD) of 100 cm, and Source to Surface Distance (SSD) of 100 cm, respectively. The electrometer bias voltage for the solid phantom setups was set at +300.0 V. The dose is defined as:

(2) $$D_{sw}\left(d_{cal}^{SW}\right)=k_{1}k_{PT}M_{sw}$$

where *k*_1_, *k*_*PT*_, and *M*_*sw*_ are the cross-calibration factor relating the dose measured in the solid water phantom to the dose measured under TG51 condition with the ADCL traceable chamber defined in Equation (4), the temperature/pressure correction factor, and the raw measurement in the solid phantom, respectively. Apply percentage depth dose, Mayneord factor, *F*, and Solid Water to water conversion factor [18], *k*^*sw*^, to Equation (2) and equating the result to (1) gives:

(3) $$k_{q}N_{w}^{c}M_{w}=k_{1}k_{PT}M_{SW}k^{sw}\text{PDD}(10)F(\text{SAD},\text{SSD})$$

Rearranging, the *k*_1_ factor can be determined as shown in (4). Apart from $N_{w}^{c}$, *k*_1_ should only be a function of setup error, and the beam quality.

(4) $$k_{1}=\frac{k_{q}N_{w}^{c}M_{w}}{k_{PT}M_{SW}k^{sw}\text{PDD}(10)F(\text{SAD},\text{SSD})}$$

To provide a metric that can be used to monitor the process across the network, a chamber independent, dimensionless, cross-calibration beam quality factor, $k_{qn}^{SW}$, was derived and shown as:

(5) $$k_{qn}^{SW}=\frac{k_{1}}{N_{w}^{c}}$$

By manipulating (3) and (4), $k_{qn}^{SW}$ can be determined as:

(6) $$k_{qn}^{SW}=\frac{k_{q}M_{w}}{k_{PT}M_{SW}k^{SW}\text{PDD}(10)F(\text{SAD},\text{SSD})}$$

For each clinical energy, the beams from all Linacs in the hospital network were used to generate a beam specific $k_{qn}^{SW}$. Statistical analysis was performed on each beam energy to obtain the mean, $\langle k_{qn}^{SW}\rangle$, and standard deviation, *σ*_*k*_. The Upper Control Limit (UCL) and Lower Control Limit (LCL) were defined as follows in each energy group:

(7a) $$\text{UCL}=\langle k_{qn}^{SW}\rangle +3\sigma_{k}$$

(7b) $$\text{LCL}=\langle k_{qn}^{SW}\rangle -3\sigma_{k}$$

### 2.1. Retrospective Analysis

The process was first applied to TG-51 data at the main site of the network from 2005 to 2018 to assess the effectiveness to monitor changes in the clinical practice and obtain an estimate of the *σ*_*k*_. In addition, there was a change in the dose calculation model for electron beams from an in-house pencil beam [19] to the commercial electron Monte Carlo algorithm (Varian, Palo Alto, CA, USA) in 2015.

### 2.2. Prospective Analysis

With the knowledge learned from the historical data, the process was applied to all the machines across the network. Implementing the workflow, a survey of all the TG-51 annuals was performed in year 0 (2019) to establish the baseline $k_{qn}^{SW}$ mean values for each energy and their corresponding control limits. Corrective actions were implemented to improve the workflow. The effectiveness of the actions was assessed based on the statistically significant change (p-value < 0.05) of the variance in year 1 (2020).

### 2.3. Chamber Control Process

As there were 29 A12 chambers in use in the network, it was desirable to mitigate the risk of using an incorrect $N_{w}^{c}$ values during the annual cross-calibration, the mean and standard deviation of the $N_{w}^{c}$ of all the A12 chambers were also calculated. Using control charts for the chamber factors, chambers whose factors exceeded appropriate upper and lower control limits were eliminated from this study. Max 4000 electrometers are routinely calibrated to be within 0.2% with each other at the ADCL.

The variance analysis of the $k_{qn}^{SW}$ and the corresponding IROC OLSD output measurements, *D*_IROC_, were performed for the year 1 dataset to determine the similarity of the variation for energy sets that has more than 10 pairs of data points. The association between the $k_{qn}^{SW}$ and the *D*_IROC_ was assessed using ranked correlation. The relationship was considered to be clinically important or significant [20] if the p-value was found to be less than 0.10 or 0.05, respectively.

## 3. Results

### 3.1. Retrospective Analysis

In this retrospective phase, a total of 833 data points over a period of 15 years were used to assess the effectiveness of the process. The $\langle k_{qn}^{SW}\rangle$ for 6 MV and 15 MV were found to be 1.003 ± 0.004 and 0.990 ± 0.007 respectively (Table 3).

**Figure 1.**
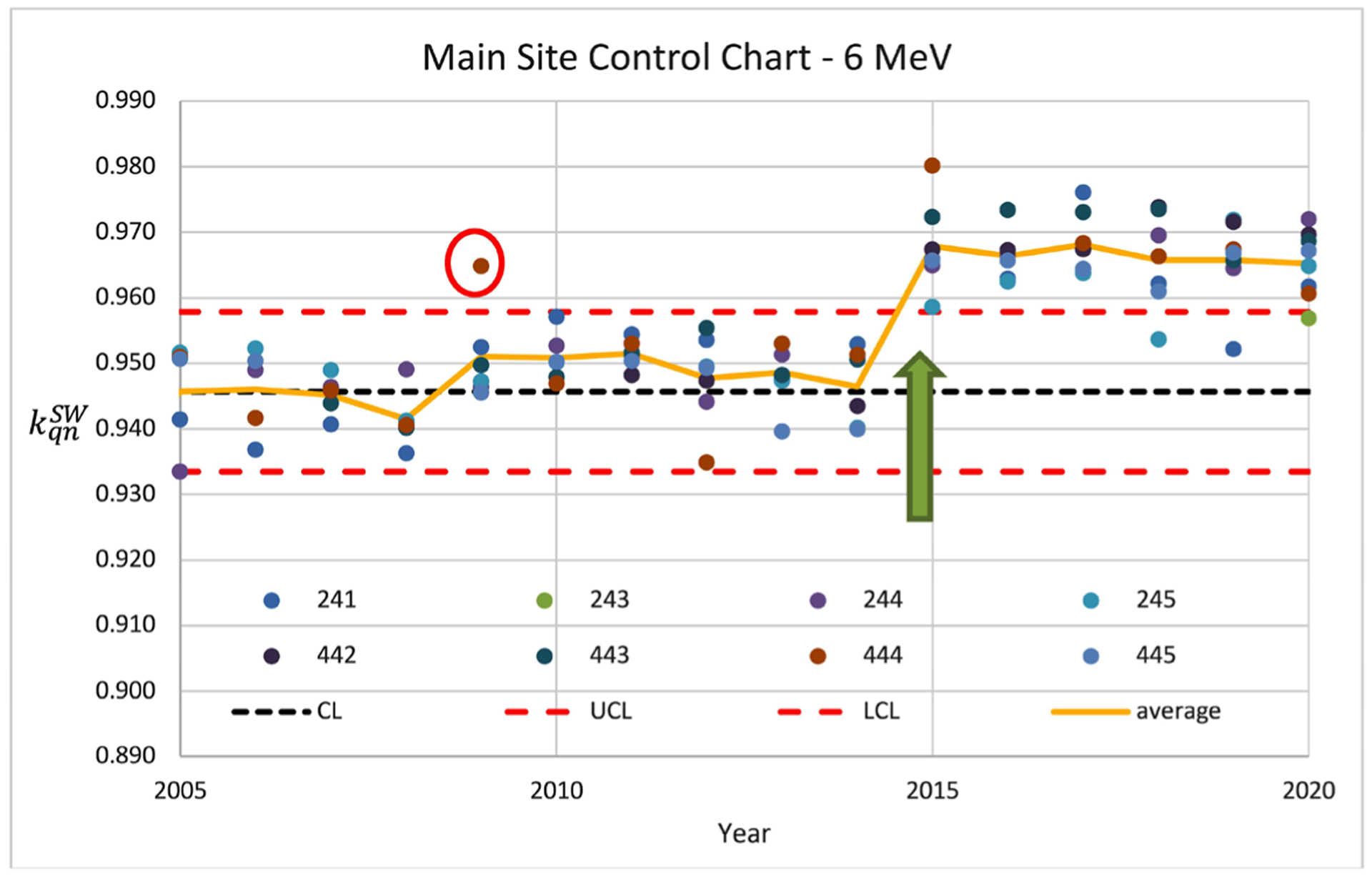
A control chart of 6 MeV electron showing the impact of algorithm change on the $k_{qn}^{SW}$ (green arrow).

For electron beams, the $\langle k_{qn}^{SW}\rangle$ were found to be from 0.953 ± 0.018 to 0.893 ± 0.013 for 6 MeV and 20 MeV. Shifts in the range of variation of $k_{qn}^{SW}$ for all electrons were observed in 2015. Figure 1 shows the control chart of the 6 MeV beam and illustrates the impact of algorithm change on the $k_{qn}^{SW}$ (green arrow).

**Figure 2.**
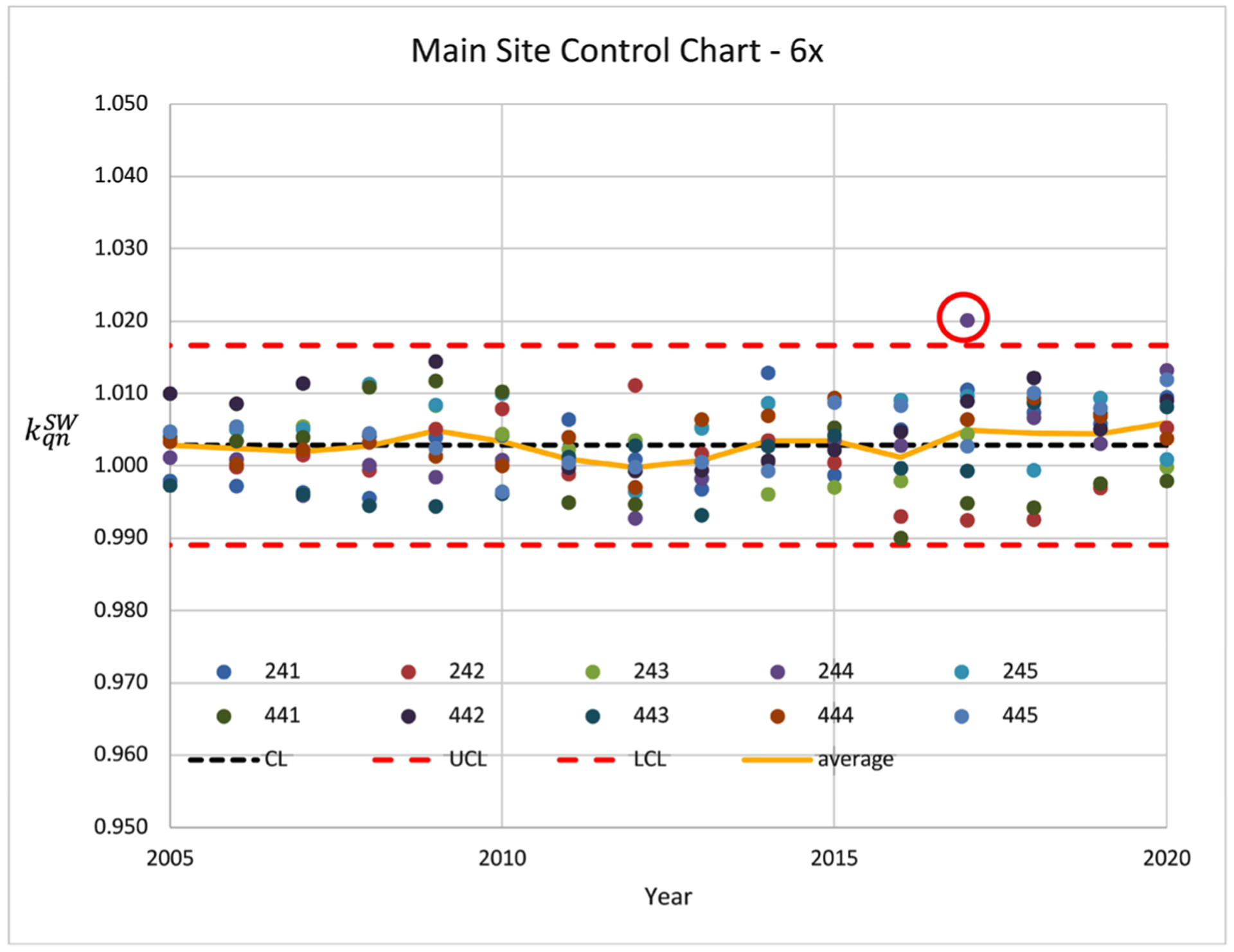
A control chart of the 6 MV photon beam from 2005 to 2020 at main site.

Similar trends were also observed in other electron energies in the same time frame. From a quality control standpoint, the control limits, CL, UCL, and LCL, should be reset at the point of the algorithm change. On occasion, individual data points exceed the control limits. One such measurement in 2009 was observed and was likely caused by setup uncertainty as the value of this machine was within the control limits in prior years. Retrospectively, this would warrant a review of the measurement point if the control chart was available at the time. Similarly, one (red circle in Figure 2) out of 169 data points was found to exceed the control limit over the 15 years and would be questionable.

To help circumvent these problem measurements, the practice at the main campus uses the average calibration factor generated from all Linacs for each energy, whereby the impacts of outliers are minimized. Interestingly, the 〈*σ*_*k*_〉 ranged between 0.3% ± 0.25% and 0.5% ± 0.25% for all energies implying a stable control limit (Table 3).

### 3.2. Prospective Network-Wide Analysis

A total of 30 machines, which comprised of the main site and six satellite centers in the hospital network, were included in this prospective phase. Table 4 shows the summary of the $\langle k_{qn}^{SW}\rangle$ and the *σ*_*k*_ for both main and the satellite sites.

Surprisingly, given the more homogeneous machine mix, the *σ*_*k*_ at the regional sites for the photon beams were found to be larger. The ranges of the control limits were found to be in the range of 1.7% – 2.6% and 3.3% – 4.2% for the main and the satellite sites, respectively. If the control limits were to be determined by using the whole network *σ*_*k*_, it would result in up to 2.0% wider control limits. Reviewing the workflows at the satellite sites, it was found that they were small variations in how TG51 and the corresponding cross-calibration were implemented; for example, the lead foil was not universally used in determining the beam quality for FFF and 15 MV beams. As a result, standardized worksheets based on the practice of the main site and the use of lead foil in determining the beam quality for FFF and 15 MV beams were implemented throughout the hospital network in year 1. The network control limits were therefore based on the tighter values from the main campus (Table 4) and would be reviewed again at the end of that year.

**Figure 3.**
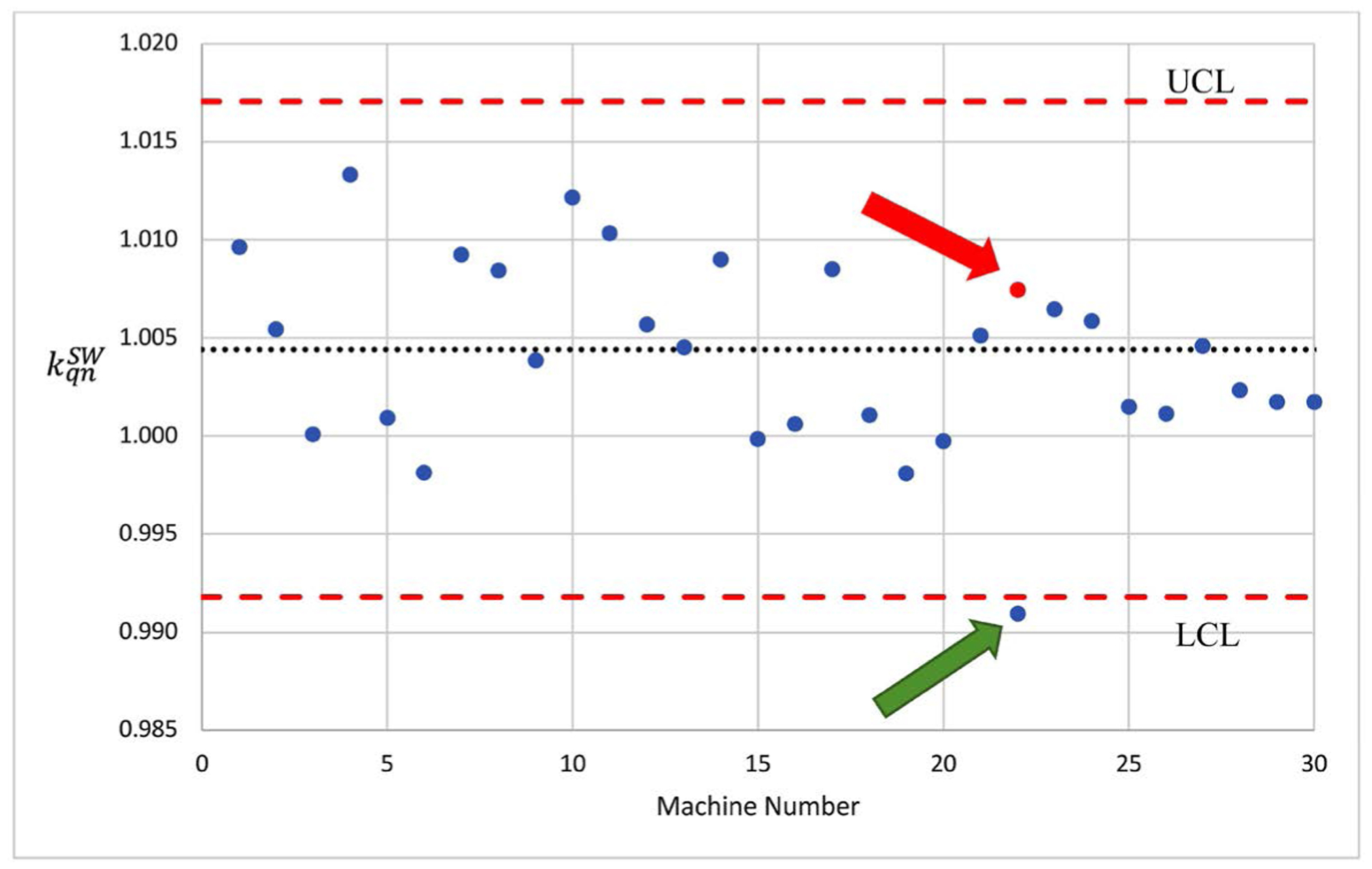
An example of using control chart for the $k_{qn}^{SW}$ to detect an out-liner (indicated by the green arrow) and the result after applying the remedial action (indicated by the red arrow) in the large hospital network.

Applying the revised baseline UCL and LCL to the TG-51 annual calibration in year 1, an outlier, which fell outside the region bound by the LCL and UCL (red dotted lines), was detected. Figure 3 shows a control chart that detected the outlier, indicated by the green arrow.

As a result, a TG-51 test was repeated with a new copy of the standard worksheet and an additional board-certified physicist on this machine. The values from the repeated measurements fell well within the control limits as shown by the red arrow and the red circle point (Figure 3). Table 5 shows the calculated $\langle k_{qn}^{SW}\rangle$ of all the energies based on all the machines in the network in year 1.

The variance analysis of each energy was performed between year 0 and 1 to determine the effectiveness of the policy change. Statistically significant reduction in *σ*_*k*_ in the satellite was observed in 6 MV and 6XFFF while no significant change in *σ*_*k*_ was observed at the main site (Table 5). The range of the control limits based on the average of all machines was found to be tightened by [0.7%, 1.7%] and [0.3%, 1.0%] for the respective photon and electron beams relative to year 0 which could be attributed to the tighter *σ*_*k*_ observed at the satellite sites in year 1 (Table 5). The *σ*_*k*_ of $k_{qn}^{SW}$ were found to be in the range from 0.42% to 0.55% for all energies indicating the reproducibility of $k_{qn}^{SW}$ for a given energy among different Linac energies was consistent. The UCL and LCL for all the energies were also shown in Table 5.

**Figure 4.**
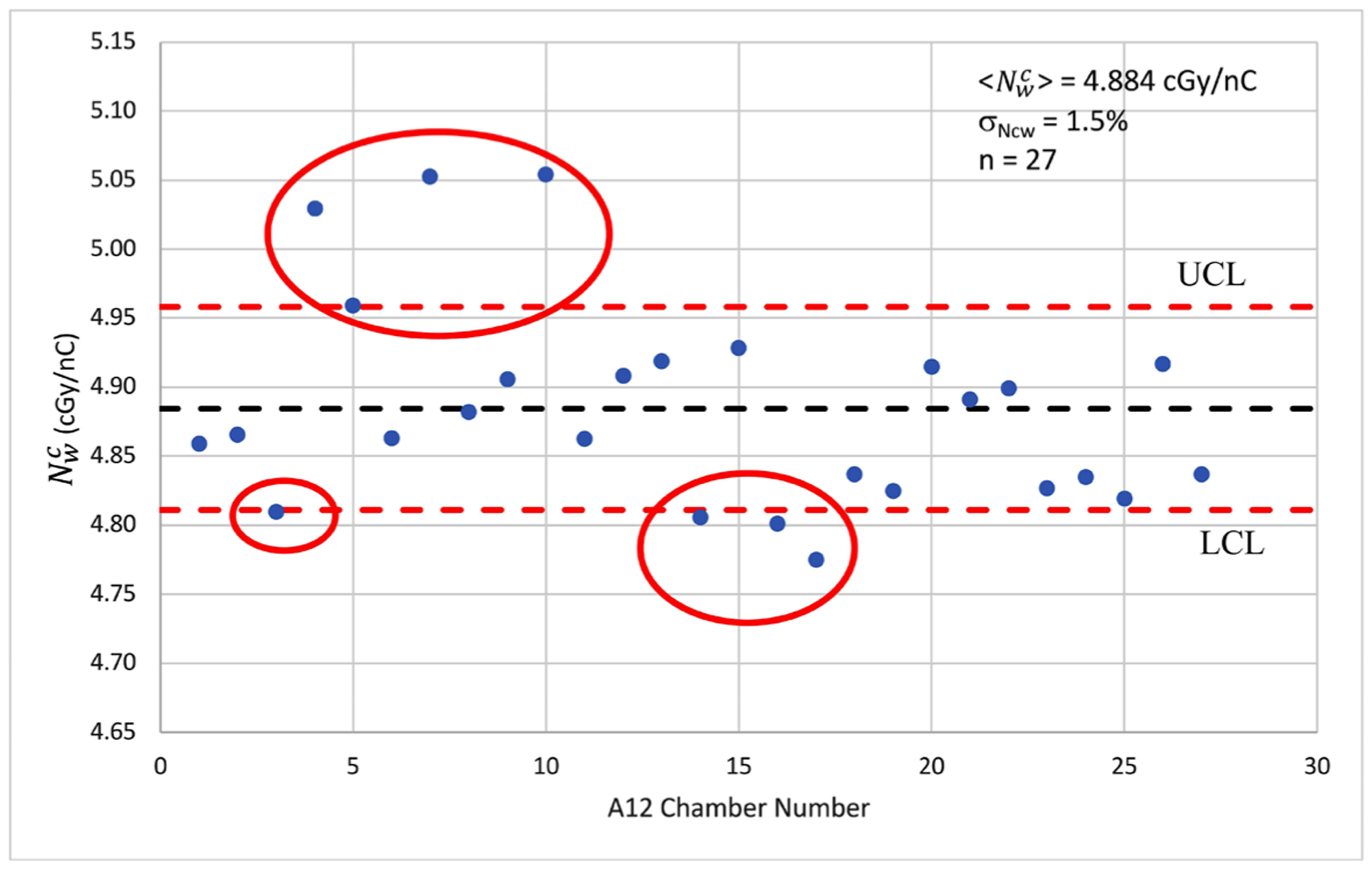
A control chart of $N_{w}^{c}$ of A12 ion chambers within the hospital network.

A total of 27-A12 farmer-type chambers were in use in the network during the two years of this study. Figure 4 shows the control chart of the chambers. The average, $\langle N_{w}^{c}\rangle$, and the standard deviation, *σ*_*Ncw*_, of $N_{w}^{c}$ were found to be 4.884 cGy/nC and 1.5% respectively.

Given the size of the *σ*_*Ncw*_ and the objective of mitigating the potential risk of using wrong $N_{w}^{c}$, the LCL and UCL was determined to be using 1*σ*_*Ncw*_ for A12. Chambers, which were found to be outside of the control limits, would only be used for monthly QA and would not be used for TG-51 annual calibration. Out of the 27 ion chambers, eight were identified (red circles in Figure 4) and taken out from the annual calibration rotation.

Regarding the IROC OSL comparison, six of the eight energies were found to have more than 10 pairs of data. The numbers of paired data were found to be between 21 to 29 for 6 MV, 6XFFF, 15 MV, 6 MeV, 9 MeV, and 12 MeV (Table 6).

The variances of $k_{qn}^{SW}$ were found to be statistically tighter than the variance of the OSLD measurements, $\sigma_{\text{IROC}}^{2}$ (Table 6). The *σ*_*k*_ was found to be about 1.7 to 3.3 times smaller than the *σ*_IROC_ indicating that $k_{qn}^{SW}$ metric has less random noise. Four of the six ranked correlation between $k_{qn}^{SW}$ and *D*_IROC_ were found to be either clinically important (p < 0.10) or statistically significant (p < 0.05) (Table 7).

## 4. Discussion

After The dimensionless energy-dependent metric, $k_{qn}^{SW}$, is derived from measurements at the time of annual calibrations at our institution’s network. By analyzing this factor with the SPC, it can be used as a metric to monitor the random and systematic variability of the reference dosimetry process throughout the network. The retrospective SPC analysis of historical data showed the effectiveness to identify a systematic change in the reference dosimetry workflow motivating to implement SPC in the prospective workflow. At the main site, the average *k*_1_, which can be taken as the proxy for $k_{qn}^{SW}$, mitigated the uncertainty in TG51 measurements. The addition of SPC, as shown in this study, provides the boundary condition when additional mitigation action is beneficial. These tools can be valuable management tools that can help clinical physicists making evidence-based risk-adjusted decisions in line with the spirit of TG-100 [9]. Prospectively, even with our relatively short period of implementation, we were able to identify a few deviations in the workflows between different hospitals in the MSK network. This feedback allowed us to channel our resources to correct specific deficiencies, in our case, lead foil usage and, to improve the consistency of our process.

Even with the best care and intention, undetectable errors, such as user error and equipment malfunction, can happen. The detected incidence shown in this study was likely caused by a combination of using the wrong calibration factor and unfamiliarity with the process. This process is able to provide quick feedback to clinical physicists about their measurements.

Reviewing the process, we realized that the $k_{qn}^{SW}$ factors out the $N_{c}^{w}$ calibration factor, which carries a range of 5.7% and a standard deviation of 1.5% for the 27-A12 chambers. After discussion among the senior QA physicists, in order to reduce the impact of potential error from using the wrong $N_{c}^{w}$, we supplemented the $k_{qn}^{SW}$ SPC metric with a much tighter 1*σ* control limit for the A12 chambers in use.

The typical standard deviation of OSLD in IROC phantom is typically within 1.6% [8] which is in line with the results found in this study. Although the *σ*_IROC_ was found to be significantly larger than *σ*_*k*_, it was interesting to find a clinical important statistical correlation between IROC OSLD and $k_{qn}^{SW}$. The correlation in this study could benefit from more longitudinal data. Further, this $k_{qn}^{SW}$ is not meant to replace the IROC OSLD process. Rather, this feedback process to the clinical physicists should be viewed as an added QA layer to the whole “Swiss Cheese” QA process [9] and a step toward the risk-based QA.

One limitation of this workflow is that the $k_{qn}^{SW}$ method was not very intuitive to clinical physicists who newly joined to the network. Proper training followed by signed off of competency is needed for all new QA physicists. As a part of the future works, data will be collected, as part of the routine annual QA test, to assess the stability and reproducibility of the $k_{qn}^{SW}$. Different techniques, such as dynamic visualization [21], will also be investigated to further explore the correlation relationship among different features of the process.

## 5. Conclusion

We have utilized SPC to monitor the pattern of Linac calibrations over 15 years and characterize the boundary conditions of the process. This allowed the prediction of annual calibration conducts across the network and the detection of any unusual events. This work has uncovered the common relationship between ADCL calibration factor of the ion chamber and Linac dose calibrations, thus identified the efficient metric that helps manage the variability in the TG-51 process. Therefore, QA physicists can be more confident in acting in a planned way before the tolerance is reached. We found that SPC coupled with a chamber independent dimensionless cross-calibration beam quality factor is a useful tool to monitor and mitigate risks in the reference dosimetry workflow of a large network.

## Figures and Tables

**Table 1.** Machine type breakdown at the main and the satellite sites.

	Main		Satellite	
Machine Type	Number	Percent (%)	Number	Percent (%)
TrueBeam	7	64%	15	79%
Trilogy	2	18%	4	21%
6EX	2	18%	0	0%
Total	11	100%	19	100%

**Table 2.** This table summarizes the calibration depth of the solid water setup (SW).

	Photon (MV)			Electron (MeV)				
Energy	6	6 FFF	15	6	9	12	16	20
*^$d_{cal}^{\text{SW}}(\text{cm})$^*	1.5	1.5	3.0	1.5	2.0	2.5	2.5	2.5

**Table 3.** This table summarizes the average and the range of the $k_{qn}^{SW}$ and *σ*_*k*_ of the beams from 2005 to 2020 at the main site.

2005–2020	Photon (MV)		Electron (MeV)				
	6	15	6	9	12	16	20
$\langle k_{qn}^{SW}\rangle$	1.003	0.990	0.953	0.945	0.930	0.906	0.893
Range	0.008	0.013	0.036	0.025	0.026	0.019	0.025
$\langle \sigma_{k}\rangle$	0.5%	0.4%	0.5%	0.4%	0.4%	0.3%	0.4%
Range	0.5%	0.5%	0.6%	0.3%	0.4%	0.5%	0.6%

**Table 4.** A summary of the baseline values $\langle k_{qn}^{SW}\rangle$, *σ*_*k*_, UCL and LCL of all the energies based on 30 machines in the hospital network.

		Photon (MV)			Electron (MeV)				
Year 0		6	6 FFF	15	6	9	12	16	20
Main	$\langle k_{qn}^{sw}\rangle$	1.005	1.018	0.995	0.967	0.952	0.938	0.907	0.897
	*σ*_*k*_	0.4%	0.3%	0.3%	0.6%	0.5%	0.3%	0.3%	0.5%
	UCL-LCL	2.6%	1.7%	1.9%	3.6%	2.6%	1.8%	1.7%	2.5%
Satellite	*$\langle k_{qn}^{sw}\rangle$*	1.001	1.014	0.990	0.964	0.947	0.932	0.903	0.893
	*σ*_*k*_	0.8%	0.6%	0.6%	0.7%	0.6%	0.6%	0.6%	0.6%
	UCL-LCL	4.6%	3.5%	3.6%	4.4%	3.9%	3.6%	3.5%	3.5%
All the machines in the network	*$\langle k_{qn}^{sw}\rangle$*	1.002	1.015	0.991	0.965	0.948	0.934	0.904	0.895
	*σ*_*k*_	0.7%	0.6%	0.6%	0.7%	0.6%	0.6%	0.5%	0.6%
	UCL-LCL	4.2%	3.3%	3.4%	4.2%	3.8%	3.5%	3.3%	3.4%
Network Baseline	UCL	1.018	1.027	1.004	0.985	0.965	0.947	0.915	0.910
	LCL	0.993	1.010	0.985	0.949	0.939	0.928	0.898	0.885

**Table 5.** A summary of the $\langle k_{qn}^{SW}\rangle$, *σ*_*k*_, UCL and LCL of all the energies based on 30 machines in the network in year 1 after implementing the standardizing of procedures.

		Photon (MV)			Electron (MeV)				
Year 1		6	6 FFF	15	6	9	12	16	20
Main	$\langle k_{qn}^{sw}\rangle$	1.007	1.018	0.997	0.966	0.952	0.939	0.908	0.900
	*σ*_*k*_	0.5%	0.5%	0.3%	0.5%	0.3%	0.5%	0.2%	0.3%
	p-value	0.234	0.07	0.414	0.412	0.239	0.128	0.24	0.09
Satellite	$\langle k_{qn}^{sw}\rangle$	1.004	1.015	0.992	0.965	0.949	0.935	0.906	0.896
	*σ*_*k*_	0.3%	0.4%	0.4%	0.6%	0.5%	0.5%	0.6%	0.5%
	p-value	<0.01	0.03	0.06	0.06	0.164	0.326	0.422	0.292
All machines in the network	*$\langle k_{qn}^{sw}\rangle$*	1.004	1.016	0.993	0.965	0.950	0.936	0.906	0.897
	*σ*_*k*_	0.4%	0.4%	0.4%	0.6%	0.5%	0.5%	0.5%	0.5%
	UCL	1.017	1.029	1.006	0.981	0.964	0.951	0.921	0.910
	LCL	0.992	1.002	0.980	0.949	0.936	0.921	0.892	0.884
	UCL-LCL	2.5%	2.6%	2.6%	3.2%	2.8%	3.0%	3.0%	2.6%

**Table 6.** The variance analysis between $k_{qn}^{SW}$ and IROC OSLD with pair data more than 10 within the hospital network.

Energy	6 MV	6 FFF	15 MV	6 MeV	9 MeV	12 MeV
N	29	21	26	24	24	26
$\sigma_{k}^{2}$	1.76 × 10^−5^	1.91 × 10^−5^	1.83 × 10^−5^	2.69 × 10^−5^	2.44 × 10^−5^	2.93 × 10^−5^
*σ*_*k*_	0.0042	0.0044	0.0043	0.0052	0.0049	0.0054
$\sigma_{\text{IROC}}^{2}$	1.12 × 10^−4^	1.73 × 10^−4^	7.82 × 10^−4^	2.14 × 10^−4^	2.67 × 10^−4^	1.40 × 10^−4^
*σ*_IROC_	0.0106	0.0132	0.0088	0.0146	0.0163	0.0119
p-value	<0.01	<0.01	<0.01	<0.01	<0.01	<0.01

**Table 7.** The ranked correlation between $k_{qn}^{SW}$ and output measured from IROC OSLD.

Energy	6 MV	6 FFF	15 MV	6 MeV	9 MeV	12 MeV
*ρ*	−0.231	−0.445	−0.331	−0.379	−0.416	−0.178
n	29	21	26	24	24	27
p-value	0.229	0.043	0.098	0.068	0.043	0.375
